# Polydopamine/Transferrin Hybrid Nanoparticles for Targeted Cell-Killing

**DOI:** 10.3390/nano8121065

**Published:** 2018-12-17

**Authors:** Daniel Hauser, Manuela Estermann, Ana Milosevic, Lukas Steinmetz, Dimitri Vanhecke, Dedy Septiadi, Barbara Drasler, Alke Petri-Fink, Vincent Ball, Barbara Rothen-Rutishauser

**Affiliations:** 1 Adolphe Merkle Institute, University of Fribourg, Chemin des Verdiers 4, 1700 Fribourg, Switzerland; daniel.hauser@unifr.ch (D.H.); manuela.estermann@unifr.ch (M.E.); ana.milosevic@unifr.ch (A.M.); lukas.steinmetz@unifr.ch (L.S.); dimitri.vanhecke@unifr.ch (D.V.); dedy.septiadi@unifr.ch (D.S.); barbara.drasler@unifr.ch (B.D.); alke.fink@unifr.ch (A.P.-F.); 2 Université de Strasbourg, Faculté de Chirurgie Dentaire, 8 Rue Sainte Elisabeth, 67000 Strasbourg, France; vball@unistra.fr; 3 Institut National de la Santé et de la Recherche Médicale, Unité Mixte de Recherche 1121, 11 Rue Humann, 67085 Strasbourg CEDEX, France

**Keywords:** cell targeting, lysosomal membrane permeabilization, polydopamine/transferrin nanoparticles, live cell imaging, targeted apoptosis in vitro, 3D cell printing, spheroids

## Abstract

Polydopamine can form biocompatible particles that convert light into heat. Recently, a protocol has been optimized to synthesize polydopamine/protein hybrid nanoparticles that retain the biological function of proteins, and combine it with the stimuli-induced heat generation of polydopamine. We have utilized this novel system to form polydopamine particles, containing transferrin (PDA/Tf). Mouse melanoma cells, which strongly express the transferrin receptor, were exposed to PDA/Tf nanoparticles (NPs) and, subsequently, were irradiated with a UV laser. The cell death rate was monitored in real-time. When irradiated, the melanoma cells exposed to PDA/Tf NPs underwent apoptosis, faster than the control cells, pointing towards the ability of PDA/Tf to mediate UV-light-induced cell death. The system was also validated in an organotypic, 3D-printed tumor spheroid model, comprising mouse melanoma cells, and the exposure and subsequent irradiation with UV-light, yielded similar results to the 2D cell culture. The process of apoptosis was found to be targeted and mediated by the lysosomal membrane permeabilization. Therefore, the herein presented polydopamine/protein NPs constitute a versatile and stable system for cancer cell-targeting and photothermal apoptosis induction.

## 1. Introduction

Over the last few years, the emerging field of nanomedicine has developed a versatile array of promising new applications, in the fields of therapy and diagnosis. A range of nanomaterials, such as liposomes, polymers, dendrimers, carbon nanotubes, and metallic nanoparticles (NPs), have been designed for drug delivery and cancer therapy [1]. Advanced functionality, including stimuli-responsiveness, is considered to be of major importance when administering medication, in a smart and targeted fashion, to a specific cell population or area, with the aim of concentrating particles in diseased cells, while reducing the systemic side effects [2,3]. In addition to their advanced functionalities, NPs can reach a wider range of cellular and intracellular targets, due to their small size and high mobility [2,4,5]. Thereby, nanotechnology is helping to tackle the increasing challenge of drug resistance that has been reported in cancer therapy—currently, the predominant impediment to an effective treatment [6].

A frequently-used approach of exploiting the advanced functionalities of nanomaterials, for cancer treatment, is the stimuli-induced increase of temperature inside cells or tissues, i.e., hyperthermia. Various stimuli can be used to this end, i.e., magnetic fields for superparamagnetic iron oxide nanoparticles (SPIONs), radio waves for silica NPs, and ultrasound or light for gold NPs [7,8,9,10]. It has been shown, in mice, that the elevation of intra-tumoral temperature to at least 42 °C, significantly enhances the vascular permeability of macromolecules, polymeric drugs, and even NPs, up to a size of 100 nm [11,12,13]. At even higher temperatures, these heat-generating stimuli can be used to induce cell death by apoptosis or even necrosis [14].

A relatively new approach to inducing hyperthermia is the induction of lysosomal membrane permeabilization (LMP) [15]. Lysosomes are the sophisticated “waste bag” of the cell, where they degrade biomolecules and recycle their individual parts, i.e., amino acids [16]. However, lysosomal leakage results in apoptosis [17]. There are a number of possibilities to induce LMP—viral proteins, reactive oxygen species, and proteinases [17]. LMP presents a promising cancer treatment target since it is not a biochemical process but a biophysical one, and therefore, is unlikely to lead to resistance. In recent work, Domenech et al. used SPIONs conjugated to the epidermal growth factor and subsequently succeeded in inducing LMP, via heat generation, using an alternating magnetic field [18].

Nanotechnology provides the means to apply the heat required for LMP and, hence, for killing cancer cells. However, NPs designed for human biomedical applications have to be investigated thoroughly towards their biocompatibility and biological fate, which can have unforeseen outcomes, especially, when the nanomaterials are found to persist in an organism [19,20]. Polydopamine (PDA), a melanin-derived polymer, has gained attention recently, due to its biocompatibility and versatility [21,22]. Melanins have interesting properties, such as heat-induction upon exposure to UV-light. The heat induction by melanins has been described as a very efficient process [23]. Most of the energy absorbed is released non-radiatively, as heat (99%), and only a low percentage is emitted radiatively (i.e., quantum yield of 0.2%) [24]. Melanins are present in a number of organisms, such as smokescreens of Cephalopods and in microorganisms, as a UV-radiation protection [25]. PDA was originally discovered in the mussel species *Mytilus edulis*, which uses the polymer as an adhesive to attach to various, usually non-reactive, surfaces [26]. Based on this, PDA was initially used as a surface modification agent, as PDA films can be formed on virtually any surface and, thereby, increase their chemical adaptability [26]. Later, Liu et al. reported the formation of nanoparticle-like materials from PDA, opening the possibilities of drug delivery and cancer treatment [27].

PDA fulfils its UV protective task in nature, by absorbing and converting harmful UV radiation energy to heat [22]. The characteristics of naturally occurring melanin make the closely-related PDA very attractive for hyperthermia applications [18]. Liu et al. injected PDA NPs, intra-tumorally, into rats and, subsequently, irradiated the exposed region by laser light, resulting in a complete disappearance of the tumor [27].

In order to overcome the need for intra-tumoral injection, which lacks precision and is effective only in the injected region, the addition of a targeting moiety, to the NPs, could improve their systemic delivery to specific cells/tissues [4,28]. The cellular trafficking of iron presents the following pathway—Transferrin (Tf), a 75.2 kDa glycoprotein, binds Fe^2+^ and subsequently attaches to the transferrin receptor (TfR), expressed on the outer cell membrane, mediating the cellular engulfment. The increased metabolic needs of fast-dividing cells (i.e., cancer cells) have been found to promote TfR expression, making this pathway a promising cancer target [29,30]. However, it must be mentioned, that the conditions, in vivo, can decisively alter the behavior of the targeted NPs [31]. When introducing NPs to complex biological fluids, the formation of a protein corona can impede the targeting [31]. For instance, it was found that the biodistribution to the cancerous tissue is not significantly increased by the addition of a targeting molecule, which could be due to a shielding of the particle surface with proteins [32]. The formation of the protein corona, however, highly depends on the NP characteristics and the method by which the targeting was grafted onto the NPs [32]. The exact mechanisms playing a role in the formation of the protein corona have not yet been fully understood, and Tf has already been successfully used for targeting purposes, on several occasions. Zhou et al. reported the design of gold/polydopamine-Methylene Blue@Bovine Serum Albumin-glutaraldehyde-Transferrin composite particles, which were used to visualize the particles inside the targeted 4T1 breast cancer cells, by surface-enhanced Raman scattering and fluorescence imaging, thus, validating both the feasibility of Tf as a targeting moiety and the biocompatibility of PDA [33]. Herein, we simplify the system using a hybrid PDA/protein NP synthesis method, recently introduced by Bergtold et al., describing the self-assembly of proteins with PDA, into the core-shell NPs [34]. Core-shell NPs have gained a considerable amount of attention due to their versatility, which can be achieved by combining a core and a shell of different materials and, therefore, combine their properties [35]. A common approach is the fabrication of a drug-loadable core and the addition of a protective shell, which can be modified by a targeting moiety. One example has been described by Malarvizhi et al. who used poly(vinyl alcohol) to assemble the core, loaded it with doxorubicin, added a layer of human serum albumin (HSA), which was also loaded with a drug (i.e., sorafenib) and conjugated the NP with Tf as a targeting agent [36]. The researchers, thereby, provided evidence for the advantages of the combination of different materials in a core-shell manner, in this case combinatorial drug delivery [36]. Ding et al. used a Fe_3_O_4_ core and SiO_2_ shell, to which they coupled doxirubicin and immobilized Tf, for targeting purposes [37]. This approach was reported to increase the efficiency of uptake, mediated by Tf, as well as an increased efficacy of the applied drug [37]. The synthesis approach for PDA/protein core-shell NPs, published by Bergtold et al., is straightforward, stable, reliable, and inexpensive. It allows for a broad variety of proteins to be incorporated into the NPs, as well as to tune the particle size by changing the ratio of proteins and dopamine. This is in contrast to the previously discussed PDA NP systems, which require the conjugation of the targeting moiety, in a stepwise synthesis [33]. To date, only bare PDA NPs were synthesized in a one-step reaction, while any additional functionalities had to be added in a separate reaction. Moreover, the simultaneous addition of the proteins provides control over the size of the synthesized NPs, resulting in a nanoparticle size dimension below 100 nm, which can be advantageous for the biomedical application [38]. We developed this system with the aim of targeting melanoma cells in vitro and inducing LMP-mediated apoptosis, via the heating properties of PDA, upon irradiation with UV-light.

## 2. Materials and Methods

### 2.1. Nanoparticle Synthesis and Characterization

PDA/protein NPs were prepared according to previously published work [34]. Briefly, the proteins, i.e., Tf [Sigma Aldrich, T0665, Buchs, Switzerland], transferrin coupled with alexa-488 [Thermo Fisher Scientific, T13342], or human serum albumin [Sigma Aldrich, A9731-5G], were dissolved in Tris-HCl-buffered water, with a pH of 8.5, at a ratio of 1:1 to dopamine [Sigma Aldrich, H8502], and stirred at 300 rpm, for 24 h. The mixture was added to a cellulose tube [Spectra/Por, 131486], with a molecular weight cutoff of 1000 kDa, and dialyzed for 24 h, to remove the unbound dopamine and protein. The sizes of the NPs, directly after dialysis but before the filtering for cell culture, were determined by dynamic light scattering (DLS) [Brookhaven 90Plus Particle Size Analyzer, Brookhaven, Holtsville, USA]. The protein content was determined by measuring the PDA/HSA NP dispersion (3.6 mg/mL) and PDA/Tf NP dispersion (3.3 mg/mL), and UV-vis-NIR spectra of the NP dispersions were measured with a UV-Vis-NIR spectroscope [Jasco V-670, Schlieren, Switzerland]. The ζ-potential was analyzed by electrophoretic mobility [Brookhaven 90 Plus Particle Size Analyzer] in H_2_O and both used a complete cell culture media (see “cell culture” below). The scanning electron micrographs were recorded using a Tescan Mira3 LM FE [Tescan, Brno, Czech Republic] scanning electron microscope. The NPs were drop-casted and sputter-coated with 3.5 nm of gold, before use.

### 2.2. Thermal Measurements via the Lock-In Thermography

A previously described lock-in imaging setup was applied to perform thermal measurements [39,40]. PDA/Tf and PDA/HSA NPs were stimulated by a homogeneous light source at 150 mA and a wavelength of 400 nm. The trigger modulation frequency was set to 1 Hz and a total of 60 cycles were conducted, for all measurements. An infrared camera [Onca-MWIR-InSb-320, XenICs, Loewen, Belgium] was mounted on a standard microscope stand [Leica Microsystems, Wetzlar, Germany] and an acquisition of infrared images was performed with a frame rate of 200 Hz. Both samples were measured five times and the average ± standard deviation was calculated.

### 2.3. Cell Culture

For the cell experiments, mouse macrophage (J774A.1) [ATCC, TIB-67TM, Manassas, VA, USA] and mouse melanoma (B16F10) [ATCC, CRL-6475] cell lines were used. The macrophages were cultured at the Roswell Park Memorial Institute (RPMI) 1640 [Gibco, 42401-018, Basel, Switzerland], supplemented with 1% L-glutamine [Gibco, 25030-024], 1% penicillin-streptomycin [Gibco, 15140-122], and 10% heat-inactivated fetal bovine serum (FBS) [ATCC, 30-2030]. For culturing the melanoma cells, Dulbecco’s Modified Eagle Medium (DMEM) [Gibco, 41966-029] supplemented with 1% L-glutamine, 1% penicillin-streptomycin, and 10% heat-inactivated FBS was used. Both cell lines were incubated at 37 °C, under a 5% CO_2_ water-saturated atmosphere, in tissue culture flasks 75 [TPP, 90076, Trasadingen, Switzerland]. For the sub-culturing of the melanoma cells, the cells were washed with phosphate buffered saline (PBS) [Gibco, 10010-015] and subsequently incubated for 5 min with trypsin-EDTA [Gibco, 25300-054], until the majority of the cells detached. For the detachment of the macrophages, a cell scraper [Sarstedt, 83.1832, Nuembrecht, Germany] was used.

### 2.4. Cell Viability Assays

Cells were seeded at a number of 20,000 cells/well (in 480 μL) into an eight-chamber well slide (0.69 cm^2^/well) [FALCON, 354108/354118, Thermo Fisher Scientific, Reinach, Switzerland] and incubated at 37 °C in 5% CO2, for 48 h. The cells were then washed with PBS and different concentrations of the PDA/protein NPs (i.e., 5 μg/mL, 10 μg/mL, 20 μg/mL, 40 μg/mL, 80 μg/mL and 160 μg/mL), in a complete cell culture medium were added, followed by a 24 h incubation, at the cell incubator conditions. To measure cell viability, two different approaches were used. (i) Lactate dehydrogenase (LDH) assay to assess the membrane rupture, and (ii) resazurin to assess the amount of viable cells with active metabolism. To this end, the supernatant was collected and an LDH assay was performed, according to the instructions of the provider [Roche, 11644793001, Basel, Switzerland], and quantified by a spectrophotometer [BioRad, Benchmark Plus, Hercules, CA, USA]. In addition, after removal of the supernatant, a cell culture medium containing 22 μg/mL resazurin, was added to the wells, incubated for 3 h, and then the formed resorufin was quantified, fluorometrically, at 590 nm [Perkin Elmer, 1420 Multilabel Counter, Waltham, MA, USA]. Based on the interference results, i.e., the same experimental setup and measurements conducted in the absence of cells, only the highest measured concentration of the PDA/Tf and the PDA/HSA NPs, respectively, resulted in increased background absorbance values. In the cell experiments, the used concentrations are significantly lower than the one which resulted in an increased background.

### 2.5. Immunofluorescent Staining

Cells were seeded at a density of 20,000 cells/well into an eight-chamber well slide. PDA/protein NPs, at a concentration of 20 μg/mL, were added to the cells. After incubation for 24 h, the cells were washed, once, with PBS and subsequently fixed with 4% paraformaldehyde [Sigma] solution, for 15 min. After another washing step with PBS, the cells were permeabilized for 15 min, using a solution containing 0.2% Triton X-100 [Fluka, 93418, Buchs, Switzerland] in PBS. Subsequently, the cells were washed, once again, and 200 μL staining solution, per well, was added. The antibody solution contained 1% bovine serum albumin (to avoid unspecific binding) [Sigma Aldrich, A7030], 0.1% Triton X-100 in PBS, antibody (anti-HSA-FITC [abcam, ab19182, Cambridge, UK]: 1:100, anti-transferrin-FITC [abcam, ab34670]: 1:100, anti-transferrin receptor-FITC [abcam, ab33996]: 1:50), DAPI [Sigma Aldrich, Switzerland], and rhodamine-phalloidin [Invitrogen, R415, Carlsbad, CA, USA]. The control stainings were performed without antibodies. After a 1 h incubation period in the dark, the plates were washed three times with PBS and finally mounted with Glycergel^®^ [Dako, C0563, Basel, Switzerland]. The analysis was carried out with a Zeiss LSM 710 confocal microscope [Axio Observer.Z1, Carl Zeiss, Germany].

### 2.6. Light-Induced Cell-Killing

Cells were seeded on cover glass bottom microscopy slides [Naige Nun International, 155382, Rochester, NY, USA] in plastic wells, with a density of 50,000/well, and incubated at 37 °C and 5% CO_2_, for 24 h. Then, the PDA/protein NPs were added to the wells at a concentration of 20 μg/mL and the slides were incubated for another 24 h. Subsequently, the supernatant containing the NPs was removed, the wells washed, once, with PBS to remove the weakly-associated NPs, and fresh cell culture media was added. In order to visualize apoptosis, fluorescent-labeled Annexin V [Roche, 11988549001, Basel, Switzerland] was added to the media, at a ratio of 1:200, allowing the binding of Annexin V to phosphatidylserine, a marker for cellular apoptosis. The slides were examined, using a Zeiss LSM 710 microscope, combined with an incubation chamber, pre-warmed to 37 °C, with a humidified 5% CO_2_ atmosphere. The time-lapse videos were recorded with a 63x objective and a pixel size of 0.439 μm × 0.439 μm. Two defined regions (162 px × 172 px) were continuously exposed to a laser beam, at either 405 nm or 633 nm wavelength. Every 6 sec an image of the whole field of view was taken.

### 2.7. Blocking of the PDA/Tf NP Uptake

A transferrin receptor (αTfR) antibody [abcam, ab25477] was diluted 1:100 and added to the wells, 30 min before exposing the cells to the PDA/Tf NPs. Subsequently, the cells were incubated with the PDA/Tf NPs, and again with the 1:100 αTfR antibody, throughout the incubation. The experiment was then performed, as described above (2.6).

### 2.8. Analysis of the Light-Induced Apoptosis Experiments

In order to obtain a comparable quantification for the different experiments, an imageJ script was written. The regions exposed to the two different laser beams, i.e., 405 and 633 nm, were analyzed individually. In brief, the area covered by cells was detected on the bright field channel, by a variance filter with radius 1. This yielded a binary image where the area with a high variance (i.e., covered by cells) received the value 1, while the rest of the region had the value 0. The binary mask, thereby obtained, was then multiplied by the green fluorescent signal (i.e., Annexin V data), thus, eliminating all signals not overlapping with the cells, due to the multiplication by 0.

### 2.9. Lysosomal Leaching Experiment

Mouse melanoma cells (B16F10) were seeded and exposed to the PDA/protein NPs for 24 h, as described above. The cells were incubated for 1 h with 50 nM Lysotracker^®^ Red DND-99 [Molecular Probes, Eugene, OR, USA], prior to the start of the light-induced cell-killing experiment. The experiment was then continued, as described above. The mean fluorescent intensity of the different regions was analyzed by ImageJ. Additionally, a region of the same size was chosen, arbitrarily, and its M.F.I. was also recorded. The third region was then used as a baseline, as there was no increased irradiation performed, but a decrease of the Lysotracker^®^ intensity was observed, possibly, due to the inherent dye-leakage or bleaching. After subtraction of the baseline, the rate was normalized and plotted, using the GraphPad Prism

### 2.10. Detection of the TfR Using Western Blot Analysis

Western blot analysis was performed according to literature [41]. In brief, mouse macrophage (J774A.1) and B16F10 cells were grown in six-well plates, at a seeding density of 1 × 10^6^ cells per well, (0.69 cm^2^) for 24 h. The cells were then washed with ice-cold PBS and scraped, after addition of Nonident P-40 lysis buffer (150 mM NaCl, 1% NP40 and 50 mM Tris pH 8), which was completed with a protease inhibitor cocktail [Sigma]. Following the lysis of the cells, the three wells were pooled and left on ice, for 30 min. The cell lysates were centrifuged for 20 min, at 12,000 rpm and 4 °C, and the supernatant was collected. The protein concentration was measured with NanoDrop [Thermo Fisher Scientific] and the samples were adjusted to the same concentration. The samples were boiled for 5 min, in a reducing Laemmli buffer, and 50 μg were loaded on to the gradient Mini–Protean^®^ TGX™ Precast Gel [Bio Rad, Hercules, CA, USA], as well as the Precision Plus Protein™ Dual Color [Bio-Rad, Hercules, CA, USA] molecular weight standards. The gel was first run at 80 V, until the samples entered the gel, and then at 120 V, using a Bio Rad electrophoresis system [Bio Rad, Hercules]. After the Sodium dodecyl sulfate polyacrylamide gel electrophoresis (SDS-PAGE), the proteins were transferred for 1.5 h, at 58 mA [Semi dry TE70X, Wigtec AG, Switzerland] onto the polyvinilidene difluoride (PVDF) membrane, under semi-dry conditions. The membrane was then blocked with 2.5% of bovine serum albumin (BSA) in tween tris buffered saline (TTBS) (20 mM Tris, 137 mM NaCl, 0.1% Tween 20), for 2 h, at room temperature, while being mixed. The blocked membrane was then treated with a primary antibody, diluted in 0.2% BSA in TTBS (1:1000 for anti-transferrin receptor antibody [Abcam 84036] and 1:2500 for anti-β-actin antibody [Abcam 8227]), and incubated, overnight, at 4 °C. The following day, the membrane was incubated with a secondary antibody (Goat Anti-Rabbit IgG HRP [Abcam 97080]) for 2 h, at room temperature, before visualization, using a luminol reagent [FluorChemE, Protein Simple, San Jose, CA, USA] as a detection staining. The optical density of the bands was estimated using a GelAnalyzer [GelAnalyzer 2010a] and the values were normalized, based on the β-actin band.

### 2.11. Spheroid Model Generation

Sterile 1.5% (*w*/*v*) alginic acid [Sigma-Aldrich, Switzerland] was dissolved in RPMI supplemented with 10% (*v*/*v*) FBS, 1% (*v*/*v*) L-glutamine, and 1% (*v*/*v*) penicillin/streptomycin, and were sterilized before utilization. As divalent cations like Ca^2+^ are required to cause the gelling of aqueous alginic acid solutions, 75 mM CaCl_2_ [Sigma-Aldrich, Buchs, Switzerland] in a complete RPMI, was prepared according to the method used by Rowley et al. and was sterile-filtered [42]. Both solutions were stored at 0–5 °C, upon utilization. B16F10 cells were cultured, as described above, and was used for the experiment, at an 80% confluency. Cells were detached, spun down, and added to the alginic acid, resulting in a concentration of 15 million cells/mL. The solution was then mixed, added to the cartridge of the 3D Discovery™ bioprinter [3DD-G01, Ref. 900 002 770, regenHU Ltd., Villaz-Saint-Pierre, Switzerland], and agitated, using a propeller, to avoid sedimentation during the printing process. Spheroids were formed by printing droplets of alginic acid and cells, into the CaCl_2_ solution. Next, spheroids were collected, spun down, and the CaCl_2_ solution was removed. Complete DMEM was then added to wash the spheroids. Here DMEM was used due to the preference of the B16F10 cells for the DMEM over the RPMI. After removal of the medium, spheroids were mixed with a collagen type I solution, which was prepared by mixing 225 μL collagen type I rat tail [3 mg/mL, Ref. A1048301, Thermo Fisher Scientific, Reinach, Switzerland] with 150 µL complete DMEM and 88.5 μL buffer solution, containing 30 μL 10× PBS, 7.5 μL 1M NaOH [Sigma-Aldrich, Switzerland], 6 μL 7.5% (*w*/*v*) NaHCO3 [Sigma-Aldrich, Buchs, Switzerland], and 45 μL 1M HEPES [Sigma-Aldrich, Switzerland]. Thirty microliter of collagen type I solution containing spheroids, was added to each well of a 4-well microscope slide, and placed in the incubator, at 37 °C, to allow gelling. After 1.5 h, 500 μL complete DMEM was added to each well, and samples were kept in the incubator, upon utilization. For the stained samples, the B16F10 cells were incubated with the VybrantTM [Thermo Fisher Scientific] cell-labeling DiD dye, in the concentration suggested by the manufacturer. After two washing steps, the spheroids were assembled as described above.

### 2.12. Light-Induced Cell-Killing Using the Spheorids

After assembly, the spheroids were exposed to 20 μg/mL NPs for 24 h. Afterwards, they were washed, once, with PBS and subsequently treated, similar to the 2D cell cultures described above, except that the 20× objective was used. This objective records with a pixel size of 1.4 μm × 1.4 μm and the used regions were sized 100 px × 100 px. The focal plane was set to the approximate center of the spheroid and light induced cell-killing was carried out. Due to the 3D structure, the signal to noise ratio of the Annexin V signal was found to be rather low and subsequent data analysis was performed—the Annexin V channel was exported, the noise was reduced by the addition of a threshold and the application of a median filter (radius = 10 px). Then the frames were analyzed by the increase of the black area, inside the irradiated area.

### 2.13. Statistical Analysis and Image Display

For illustration purposes and the performance of student’s T tests, GraphPad Prism version 6.00 for Windows, [GraphPad Software, LaJolla, USA] was used. Imaris [Bitplane, Version 7.4.2, Belfast, UK] was used for the renderings of the confocal data sets and Huygens [SVI, Version 4.4, Hilversum, The Netherlands] was used for deconvolution.

## 3. Results and Discussion

### 3.1. Nanoparticle Synthesis and Characterization

**Figure 1:**
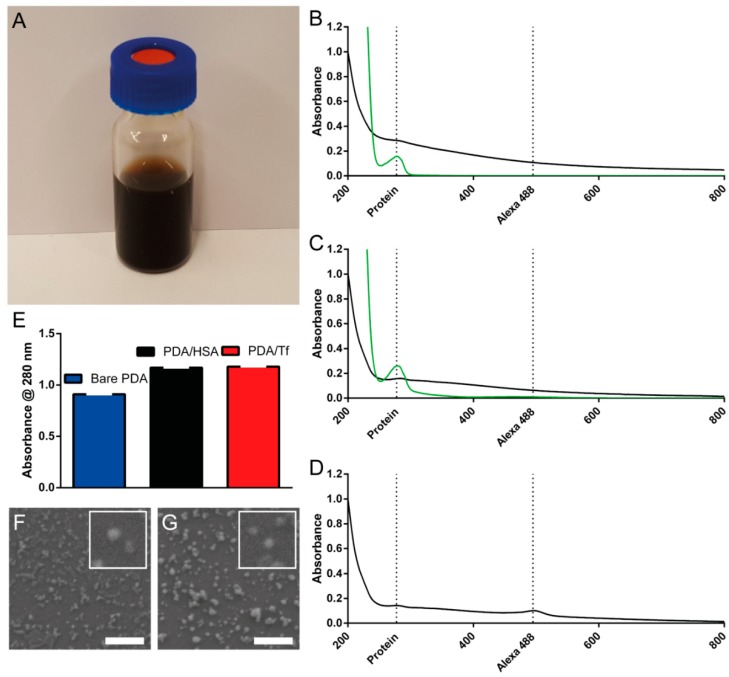
Characterization of the polydopamine particles, containing transferrin (PDA/Tf), polydopamine/human serum albumin (PDA/HSA), and PDA/Tf-A488 nanoparticles (NPs). (**A**) Photograph of an aqueous suspension of PDA/HSA NPs, and (**B**) absorbance spectra of the PDA/HSA with the subtracted background of bare PDA NPs (black line), and absorbance spectra of the HSA alone (green line). (**C**) The absorbance spectra of the PDA/Tf with subtracted background of the bare PDA NPs (black line), absorbance spectra of the Tf alone (green line), and (**D**) the absorbance spectra of the PDA/Tf-A488 (black line). The dotted lines represent typical absorbance maxima for the proteins (270–280 nm) and the Alexa 488 fluorophore (495 nm), in panels (**B**,**C**). (**E**) The absorbance measurement of the bare PDA NPs, PDA/HSA NPs, and PDA/Tf NPs, at 280 nm. The difference in absorbance of the bare PDA NPs and the PDA/protein NPs can be attributed to the proteins present. (**F**) Scanning electron micrograph of the drop-casted PDA/Tf NPs, and (**G**) scanning electron micrograph of the drop-casted PDA/HSA NPs. Scale bar = 500 nm.

For this study, three different PDA/protein hybrid particles were synthesized, i.e., PDA/HSA, (Figure 1A), PDA/Tf, and PDA/Transferrin-Alexa 488 (Tf-A488) NPs. Transferrin was chosen because of its ability to target cancer cells that heavily express TfR on their outer cell surface. The transferrin-Alexa 488 conjugate was used to observe the NP uptake via fluorescence, into the cells, in real-time and human serum albumin (HSA) served as the control protein. All the NPs were synthesized, as previously described by Bergtold et al. [34]. The hydrodynamic diameters and ζ-potential measurements for all particles are summarized in Table 1. Dynamic light scattering (DLS) data revealed a hydrodynamic diameter of PDA/Tf and PDA/HSA, in the same size regime, but a significantly larger diameter for the PDA/Tf-A488 (Table 1). This can be explained by the commercial origin of the Tf-A488 conjugate, which is sold containing a variety of proteins that are not specified by the distributor.

The mean hydrodynamic diameters of the NPs in water, were determined by DLS. Furthermore, the ζ-potential was measured in water and both of the media were used in the study. Since the size of the NPs is based on the ratio of the proteins to dopamine, in the reaction mixture, the unspecified proteins in the mixture make a size-controlled synthesis impossible [34].

As reported by Jiang et al., we assumed—due to the stochastic nature of the synthesis—an even distribution of the protein on the NP surface [35]. The slight size increase of the PDA/HSA NPs could be attributed to differences in the surface structures—or more precisely the accessibility of the synthesis-driving lysine/glutamic acid amino acid pair—between the HSA and the Tf [34]. In terms of uptake, the difference could be assumed to be negligible, since it was found that the uptake was similar for particles above or below the optimal 40 nm in diameter [35]. The ζ-potential was measured in water and the complete cell culture media (DMEM and RPMI), and was found to be negative for all NPs (Table 1). UV-Vis absorbance spectra were recorded for all NP types (Figure 1B–D). Additionally, the absorbance spectra of the HSA (Figure 1B) and the Tf (Figure 1C) were measured. To ascertain that the measured mixture did not contain free protein, all of the PDA NPs were dialyzed, as described in the report of Bergtold et al. [34]. All measured samples containing proteins revealed a small peak at a λ of 270–280 nm, which corresponded to the bound proteins HSA and the Tf [43]. In the spectra of the bare PDA NPs—which were produced as a control—this peak was not found. The particles were produced without the size control by proteins, as reported by Bergtold et al. [34]. An additional peak could be detected at a wavelength of approximately 480 nm for Tf-A488, which corresponded to the reported absorbance of the Alexa 488 dye [44]. To investigate the ratio of the PDA to proteins present in the NPs, the absorbance of the NPs at 280 nm, was measured (Figure 1C). As absorbance is additive, and the strength of light scattering compared to light absorption is expected to be negligible in this particle-size range, the difference in absorbance between the bare PDA NPs and the PDA NPs containing proteins, may be attributed dominantly to the protein part of the NPs [45]. Here, we measured a difference of 0.26 for the PDA/HSA NPs and 0.27 for the PDA/Tf NPs. Using a standard curve (Figure S1), the protein contents were determined to be 0.5 mg/mL for the PDA/HSA and 0.2 mg/mL for the PDA/Tf. These numbers corresponded to an approximate percentage of 6 for the PDA/Tf and 14 for the PDA/HSA. The shape of both the PDA/Tf (Figure 1D) and the PDA/HSA (Figure 1E) were determined by scanning electron microscopy. It was observed that especially the PDA/HSA NPs, aggregated upon drop-casting. Nevertheless, the NP size and shape were apparent. The stability of the PDA/Tf NPs was assessed by time-resolved DLS, whereby, no significant aggregation was observed during the first hour and after 24 h (Figure S2). Additionally, Chassepot et al. assessed the stability of similar NPs, over a time period of 3 months and found no significant change [46]. Together, the data confirmed the synthesis of stable PDA/HSA, PDA/Tf-A488, and PDA/Tf hybrid NPs, thus, providing defined NPs for the uptake studies.

### 3.2. Transferrin Receptor Expression in Melanoma Cells and Macrophages

Two different mouse cell lines were used in this study—B16F10 melanoma cells and J774A.1 macrophages. The J774A.1 cells were chosen because they are professional phagocytic cells and the B16F10 melanoma cancer cells because they express a high ratio of transferrin receptors on their surface. The expression level of the transferrin receptor, in both cell lines used in this study, was probed, using immunofluorescent staining and western blot analysis. First, the presence of TfR was confirmed by western blot analysis, where the TfR specific band was detected in lysates of both cell lines, as well as in the additionally included non-mitotic control—primary macrophages derived from human buffy-coat-isolated monocytes (MDM) (Figure S3). The MDMs were found to express TfR in a similar range as the J774A.1 cells. Mitotic cells are known to have an increased metabolic activity, resulting in an upregulated transferrin receptor [47,48]. B16F10 melanoma cells expressed more TfR than the J774A.1 mouse macrophages. One of the hallmarks of cancer is uncontrolled proliferation, thus, malignant cells generally exhibit elevated expression of the receptor [49,50]. Moreover, in both cell lines, the transferrin receptor could be observed, intracellularly, and on the outer cell border, by confocal laser scanning microscopy (cLSM), indicating the expression of the transferrin receptor (Figure S4). Therefore, the chosen cell systems are suitable for comparing the targeting properties of the PDA/Tf and the PDA/HSA NPs.

### 3.3. Uptake and Cell Viability

The cellular uptake of the PDA/protein hybrid NPs was analyzed by immunofluorescent staining. The mouse macrophages (J774A.1) and mouse melanoma cells (B16F10) were incubated with the PDA/HSA and the PDA/Tf NPs, for 24 h. cLSM revealed the intracellular localization of fluorescent antibodies, against HSA or Tf, in the NPs (Figure S5). The fact that an antibody against the incorporated protein was able to bind, indicated that the targeted epitope was still accessible on the surface of the PDA particles. The two particle types were detected in both J774A.1 and B16F10 cells.

The PDA/Tf-A488 NPs were administered to the J774A.1 mouse macrophages, in a live cell imaging experiment—the dynamics of the NP uptake process were assessed by measuring the fluorescence signal over time. Many particles were detected inside the cells, within hours (Figure S6 and Supplementary Video 1), which was in line with the results from the immunofluorescence experiments as the macrophages, being professional phagocytes, usually internalize any NPs deposited on the surface [51].

To measure the cell viability after exposure to NPs, J774A.1 and B16F10 cells were exposed to the PDA/Tf (Figure S7) and the PDA/HSA NPs (Figure S8) at 5, 10, 20, 40, 80, and 160 μg/mL, for 24 h, followed by a lactate dehydrogenase (LDH) assay and a resazurin test. No cytotoxicity or proliferation impairment was detected for either combination of NPs and cell lines. This might make the PDA NPs, non-toxic representatives of carbon-based NPs, for photothermal applications. Long-term toxicity of carbon-based nanomaterials is a subject of ongoing debate and, undoubtedly, needs further exploration. Nevertheless, a small number of functional, carbon-based nanomaterials have been successfully tested for phototherapeutic applications [52]. Most of the carbon-based systems explored to date, do not employ active targeting, which would potentially benefit those materials on their way to the clinic [52].

### 3.4. Heating Potential of the PDA/Tf and the PDA/HSA NPs

**Figure 2:**
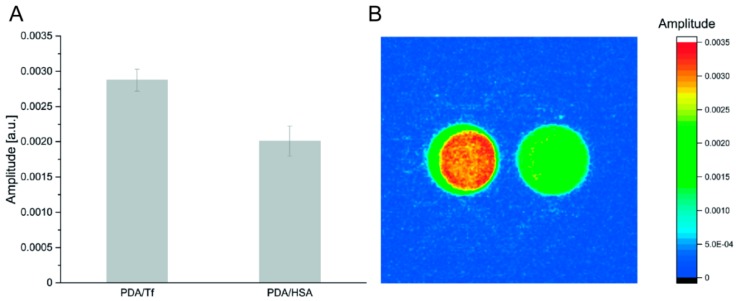
Comparison of the heat dissipation of the PDA/TF and the PDA/HSA, at 100 μg/mL (**A**). 2D amplitude heat map of the same measurement, recorded with lock-in thermography (LIT) (PDA/Tf left, PDA/HSA right) (**B**). A higher amplitude signal corresponds to a more intense heat generation. *N* = 5.

Lock-in thermography (LIT) has been used to analyze the heat generation of the PDA NPs [53,54]. This method offers a precise and sensitive means to resolve the differences between the heating capacities of the two NPs, as it allows the detection of temperature deviations in the μK range. Figure 2 shows a comparison of the PDA/Tf and the PDA/HSA, at a concentration of 100 μg/mL. This particular concentration was chosen, due to the limitations of the LIT instrument, i.e., lower concentrations did not yield enough signals for a reliable measurement. It can be seen that the Tf-functionalized particle type, generated more thermal energy than the HSA type. A possible explanation for this observation is simply the size difference (30 nm vs. 73 nm) between the measured particles. However, determination of the relationship between size and heating capacity of the PDA/protein NPs should be subject to further investigation, and is outside the scope of this study.

### 3.5. Light-Induced Apoptosis

**Figure 3:**
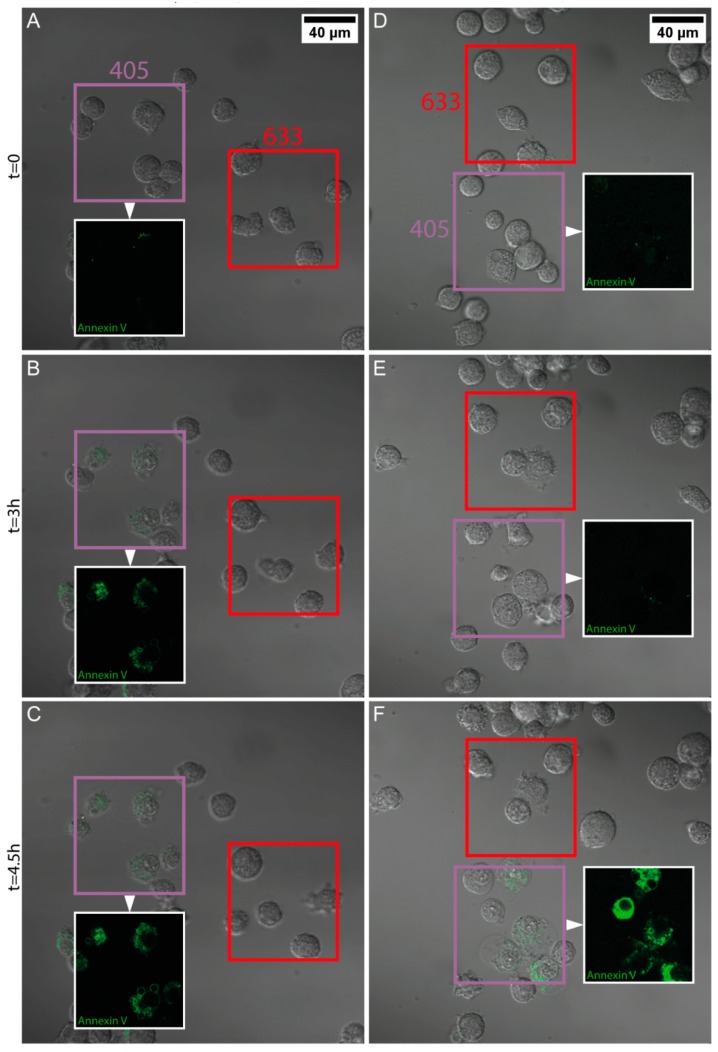
Rate of apoptosis induction in the J774A.1 mouse macrophages exposed to the PDA/HSA NPs, upon light irradiation. The macrophages were exposed to 20 μg/mL PDA/HSA NPs (**A**–**C** and Supplementary Video 2) or media only (**D**–**F** and Supplementary Video 3). Subsequently, two separate regions were defined in the field of view; one was irradiated with UV-light (405 nm, purple square), and the other with red light (633 nm, red square). The addition of the Annexin V stain (green fluorescence) allowed for real-time visualization of the onset of apoptosis. Snapshots taken throughout the duration of the experiment are presented, i.e., at t = 0 h (A and D), t = 3 h (**B**,**E**), and t = 4.5 h (**C**,**F**). For better visibility, only the Annexin V signal channel in the regions irradiated with UV-light is shown (insets).

**Figure 4:**
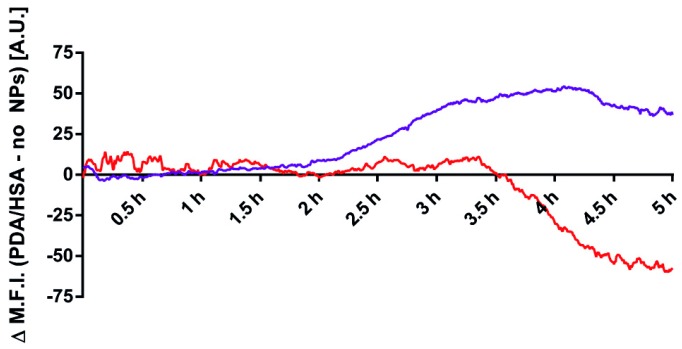
The Δ mean fluorescent intensity (M.F.I.) of the green fluorescent signals shown in Figure 3 are presented as the normalized mean fluorescent intensity, per region, and the values from the negative control results were subtracted.

**Figure 5:**
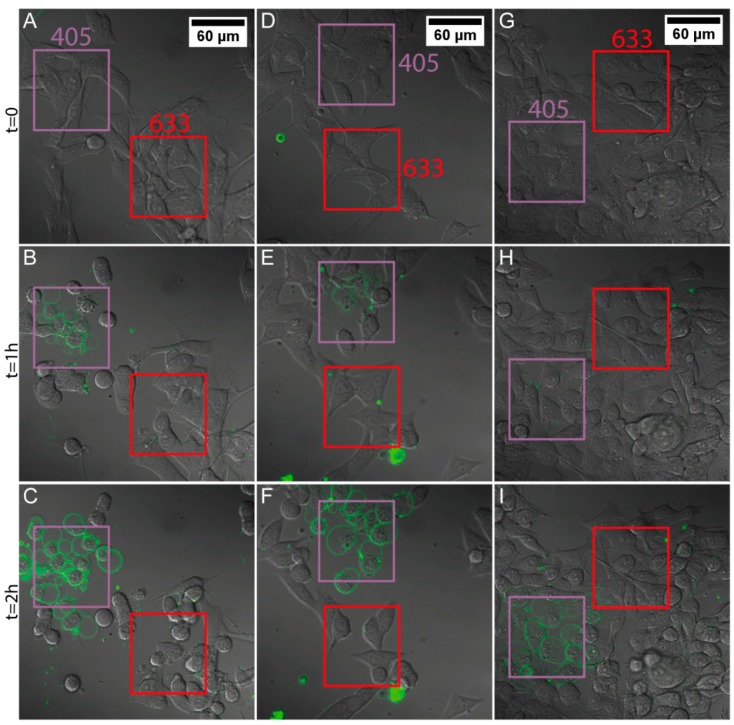
Rate of apoptosis induction in the B16F10 mouse melanoma cells exposed to the PDA/protein NPs, upon light irradiation. The melanoma cells were exposed to 20 μg/mL PDA/Tf NPs (**A**–**C** and Supplementary Video 4), PDA/HSA NPs (**D**–**F** and Supplementary Video 5), or media only (**G**–**I** and Supplementary Video 6). Similar to Figure 3, two regions, per field, were defined, one irradiated by 405 nm light (purple square) and one with 633 nm light (red square). The addition of the Annexin V dye allowed for the analysis of the apoptosis onset. Different time points are presented: t = 0 (**A**,**D**,**G**), t = 1 h (**B**,**E**,**H**), and t = 2 h (**C**,**F**,**I**). All datasets have an n value of at least three.

In order to assess the ability of the PDA hybrid NPs to exert their heating potential inside cells and, thereby, induce apoptosis, the following experiment was conducted. Both cell types were exposed to either PDA/HSA or PDA/Tf NPs, at a concentration of 20 μg/mL, for 24 h, and then observed by cLSM. Two regions of interest were excited, with either 405 nm (purple boxes in Figure 3 and Figure 5) or 633 nm (red boxes in Figure 3 and Figure 4). The laser power for the 405 nm laser was found to be 4.1 mW, whereas the laser power for the 633 nm laser was measured at 1.1 mW. Annexin V was added to the cell culture media to visualize apoptosis, since it binds to phosphatidylserine, which is only present on the outside of a cell, when the apoptotic pathway is induced [55].

**Figure 6:**
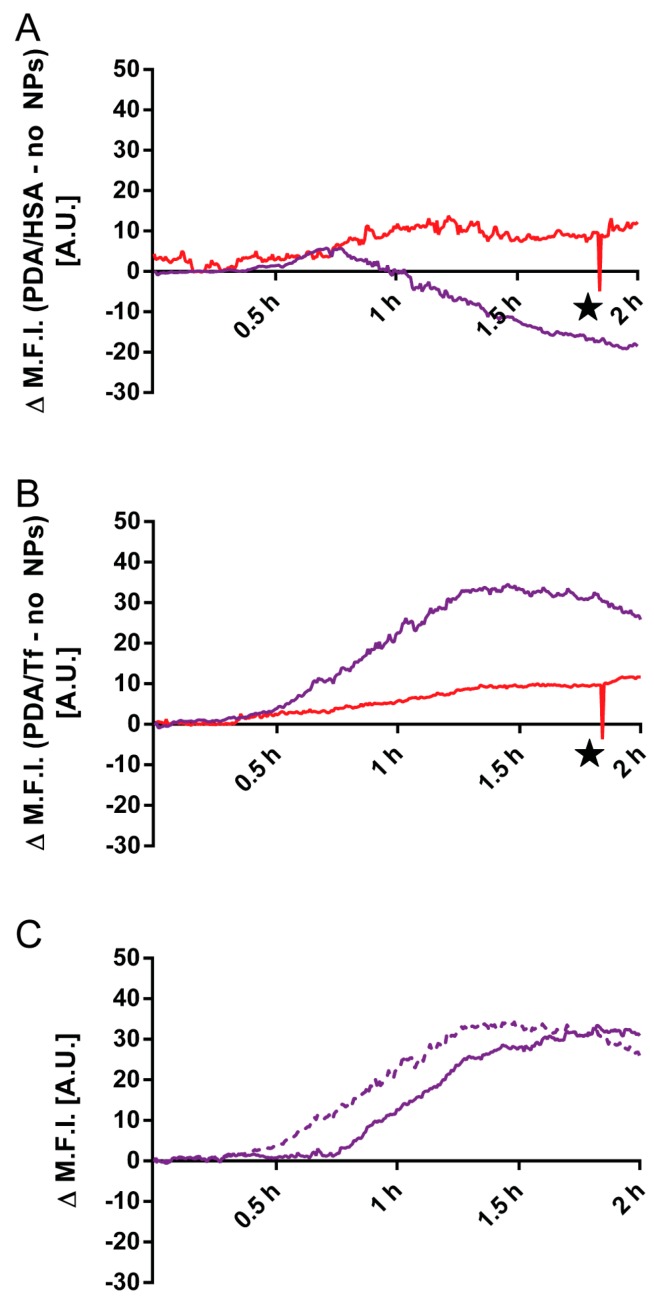
Analysis of the apoptosis rate in the B16F10 mouse melanoma cells, by UV-light irradiation dependent on NP exposure. The rate obtained for the cells not exposed to NPs was subtracted from the rate measured when the cells were exposed to the PDA/HSA NPs, for both regions, independently, yielding the Δ mean fluorescent intensity (M.F.I.) value (**A**). For the 405 nm results, a negative trend could be observed, whereas, the cell death rate in the 633 nm region increased as expected. Additionally, the effect of PDA/Tf NPs versus the negative control was evaluated by subtracting the negative control from the PDA/Tf NP data (**B**). The apoptosis rate of cells exposed to the PDA/Tf NPs was faster than the negative control, manifesting itself in an increasing ΔM.F.I. Finally, the ΔM.F.I. of the 405 nm regions of PDA/Tf vs. the negative control (dotted line) and the PDA/Tf vs the PDA/Tf blocked (solid line) were compared (**C**). There is an observable shift of the effect to a later time-point, indicating a successful blocking of the transferrin receptor. ★ The sudden drop in the 633 nm signal was due to a faulty recording in one of the frames of the repetitions. *N* = 3–4.

It was found that J774A.1 mouse macrophages exposed to 20 μg/mL PDA/HSA NPs, underwent apoptosis faster than the non-particle-exposed cells, in both regions illuminated with wavelengths of 405 nm and 633 nm (Figure 3 and Figure 4, Supplementary Videos 2 and 3). Melanoma cells exposed to the PDA/HSA NPs showed a rate of cell death equal or even lower than the negative control (Figure 5, Figure 6A and Supplementary Videos 4–6), whereas, the melanoma cells incubated with the PDA/Tf, exhibited a high degree of apoptosis induction (Figure 5B). The observed difference was thought to be due to the different uptake rates of the PDA/HSA and the PDA/Tf NPs. Tf-mediated uptake is known to be clathrin-dependent with Tf residing in early endosomes [56,57]. Usually, Tf is recycled to the cell membrane before the fusion of lysosomes with the early endosomes. The recycling of Tf takes place by pinching-off tubules with narrow diameters, while the bulk of the early endosomes matures into late endosomes/lysosomes [58]. In this case, an increase in the apoptosis rate was observed because the Tf was not free but was combined with the PDA NPs. This was expected to be a size-dependent effect—as the recycling mechanism involved small-diameter tubules, 40 nm NPs could be simply too large to pinch them off, and were thus, transported into the late endosomes, with the bulk. This enabled the PDA/Tf NPs to elicit LMP and accelerate the apoptosis. PDA/HSA NPs are not expected to enter the cells to the same degree as the PDA/Tf NPs, as their uptake mechanism might be different. The reduced rate of cell death observed was, thus, hypothesized to be due to more PDA/HSA NPs adhering to the outer cell membrane, where they also absorb UV-light and, thereby, protect the cells. UV-light itself was reported to harm mammalian cells when irradiating with energy higher than a certain threshold [59]. However, the samples with the PDA/Tf NPs present, exhibited a quicker onset of apoptosis than the negative control without any particles, which cannot be explained by the effect of the UV-light alone. Outside the defined regions, there were also single cells which underwent apoptosis (Figure 3 and Figure 5). This could be attributed to normal cellular behavior, as cells can also undergo apoptosis, when in culture, for various reasons, such as DNA damage or faulty signaling.

To prove that the PDA/Tf NPs are taken up via the Tf-receptor-mediated uptake, the PDA/Tf NPs were co-incubated with a transferrin-receptor-blocking antibody, to inhibit the NP uptake. In this experiment, the boost in apoptosis induction, observed previously, was strongly attenuated (Figure 6C). These findings provide further evidence for active-targeting and the persistence of protein activity in the PDA particles.

### 3.6. Mechanism of the Apoptosis Induction

**Figure 7:**
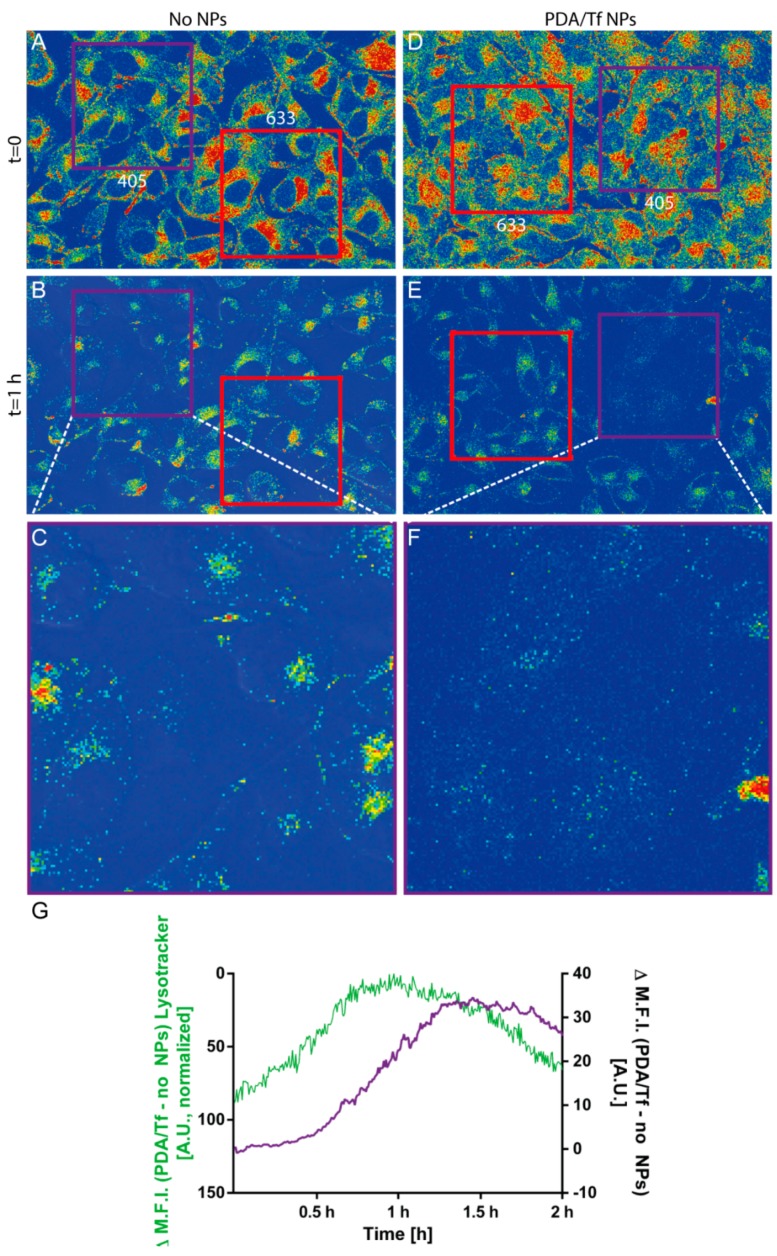
Visualization of the lysosomal marker in the B16F10 mouse melanoma cells exposed to no NPs (**A**) or the PDA/Tf NPs (**B**), and then irradiated with 405 nm and 633 nm light. After 1 h, less fluorescence of the lysosomal marker was observed in the cells exposed to the PDA/Tf NPs, irradiated with 405 nm light, than those exposed to the 633 nm light (**E**,**F,** and Supplementary Video 7), as well as to the non-particle-exposed cells (**B**,**C** and Supplementary Video 8). The decrease in the fluorescence signal of the Lysotracker^®^ was analyzed and plotted together with the signal corresponding to the apoptosis induction, in cells exposed to the PDA/Tf NPs, and irradiated with the UV-light (**G**). *N* = 3.

As stated before, the PDA/Tf NPs were thought to trigger the LMP and, thus, activate apoptosis. In order to carry out their function, the PDA/Tf NPs have to traffick into the lysosomes upon cellular uptake by receptor-mediated endocytosis. cLSM experiments using the Lysotracker^®^ and the anti-Tf antibodies, to stain the particles, revealed a significant co-localization between the lysosomal and the NP stain (Pearson’s R 0.38 for PDA/Tf NPs and the Lysotracker vs. 0.24 for the anti-Tf antibody and the Lysotracker^®^, without the presence of the NPs), indicating the presence of the PDA/Tf NPs inside lysosomes (Figure S9). In a further experiment, the signal of a Lysotracker^®^ dye measured by the cLSM, over time, was used as a metric for lysosomal instability, during light-induced cell-killing experiments in the B16F10 melanoma cells exposed to the PDA/Tf NPs. A decrease of the intensity of the Lysotracker^®^ signal was indeed observed before the apoptosis signal was observed (Figure 7, Supplementary Videos 7 and 8) and this effect was only seen when the cells were exposed to the PDA/Tf NPs. In order to remove the interfering phenomena (i.e., quenching/bleaching) from the analysis, the values of the negative control was always subtracted from the experimental data, as a baseline. These results suggest that LMP is a major driving force for apoptosis upon heat induction, and is caused by the PDA/Tf NPs.

### 3.7. Light-Induced Apoptosis in an Organotypic Melanoma Spheroid Model

**Figure 8:**
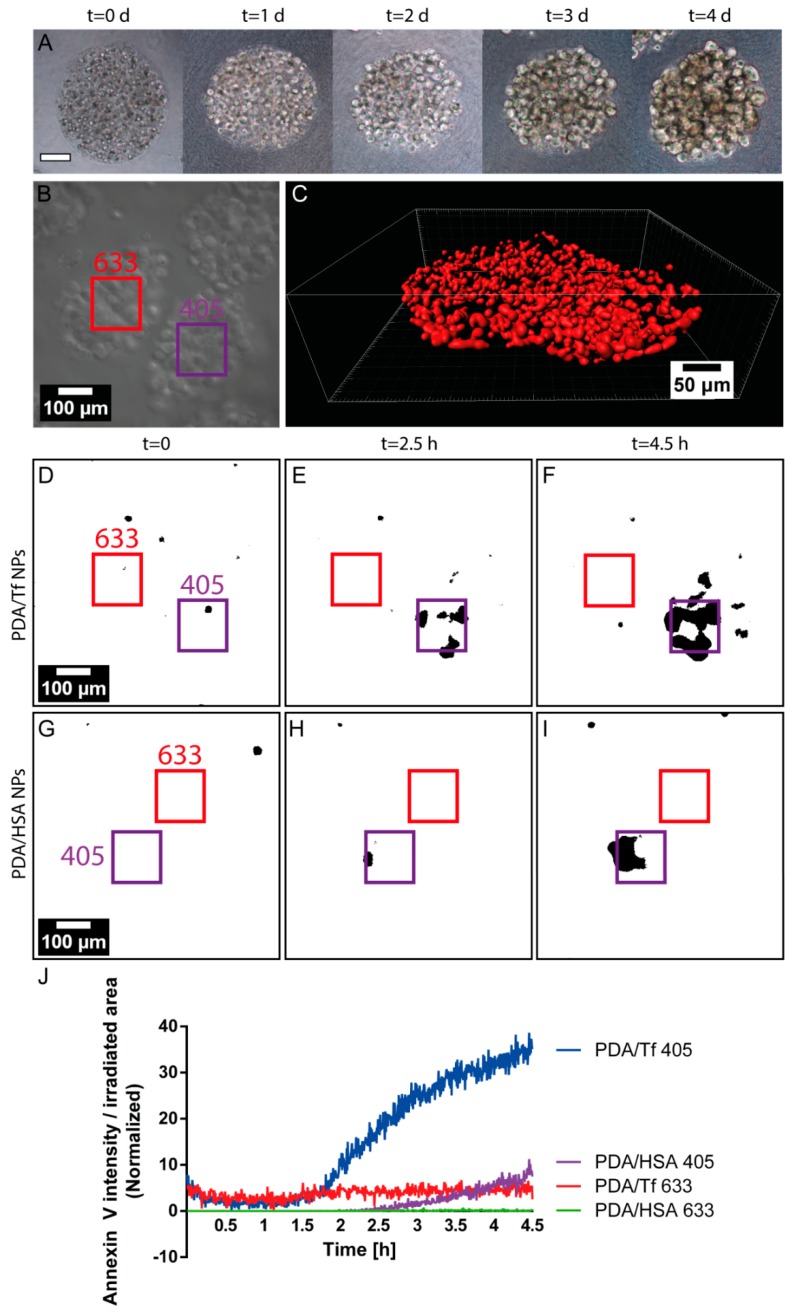
Light-induced apoptosis using melanoma spheroid models. The development of the spheroid model is depicted in the representative images in panel (**A**), corresponding to the increasing time-points during their growth. Representative phase contrast image of the spheroid model with the selected regions of elevated irradiation with 405 nm (violet) and 633 nm (red) laser light (**B**). Rendered cLSM z-stack of the stained B16F10 assembled into the spheroid model, after 24 h of development (**C**). Analyzed frames of the videos showing the B16F10 spheroids, exposed to the PDA/Tf NPs, at time point 0 (**D**), after 2.5 h (**E**), and 4.5 h (**F**), and spheroids exposed to the PDA/HSA NPs, at time point 0 (**G**), after 2.5 h (**H**), and 4.5 h (**I**). Every frame of the videos was analyzed and the Annexin V intensity per irradiated area, either 405 (violet) or 633 (red), was recorded and is shown in panel (**J**). The blue line represents the cell-death rate of the samples treated with the PDA/Tf NPs, the violet line is that of the samples treated with the PDA/HSA NPs, and both were irradiated with a 405 nm laser light. The red line represents the cell-death rate of the samples treated with the PDA/Tf NPs, the green line shows the samples treated with the PDA/HSA NPs (both irradiated with 633 nm laser light). N of PDA/Tf = 2 and N of PDA/HSA = 3.

To obtain a better understanding of the effect of the PDA NPs on the mouse melanoma cells in a more complex in vitro model, an organotypic melanoma spheroid model, mimicking the 3D microenvironment of melanoma tumor cells, was used. Spheroids of the mouse melanoma cells were prepared by mixing alginic acid with the B16F10 mouse melanoma cells and bioprinting them into a CaCl_2_ solution, which caused gelling of the alginic acid. This technique was shown elsewhere and adapted for the 3D Discovery™ bioprinting instrument, which was used for this experiment [60]. Prepared spheroids were then mixed with a collagen type I solution and the droplets containing spheroids were placed in the incubator, to allow the crosslinking of the collagen, thus, embedding the spheroids into the collagen matrix. The development of the spheroids was followed by phase contrast imaging, every day, for 4 days (Figure 8A). For visualization purposes, the spheroids were incubated with a cell tracker fluorescent dye and, after 24 of incubation, imaged, using the cLSM system. The spheroids exhibited an oval, but 3D shape (Figure 8A–C) and were able to keep their 3D structure, after 24 h incubation. The, thereby assembled spheroids, were then exposed to the PDA/HSA and the PDA/Tf NPs, for 24 h, and light-induced cell-killing experiments were performed, as described, for the 2D cell cultures. Although the onset of apoptosis was found to be at a later time-point than in the 2D cell culture model, the observed cell-death rate of the PDA/Tf NPs was again higher, compared to the PDA/HSA (Figure 8D–J). Without the presence of either the PDA/HSA or the PDA/Tf, the onset of cell death was found to be in-between, which was later than that in the samples treated with the PDA/Tf, but earlier than that in the samples exposed to the PDA/HSA (Figure S10). The time-point of the apoptosis onset in the spheroid model was, thus, well in line with the observations made in the 2D cell model, where the PDA/Tf NPs were able to increase and the PDA/HSA NPs were able to decrease the cell rate, with respect to the untreated controls, respectively. Through this experiment, a higher efficacy of the PDA/Tf NPs to induce apoptosis in a 3D organotypic melanoma spheroid model, could be shown, and future experiments with melanoma cells from patients would also offer an effective platform to test the, herein described particles, in different tumor progression stages.

## 4. Conclusions

In this paper we reported the functional design of the PDA hybrid, core-shell NPs, with different proteins. Not only was the synthesis simple and fast, the necessary components were also inexpensive. The synthesized NPs were biocompatible, even at high concentrations (160 μg/mL), in vitro. The NPs retained the protein functionality at the surface, as shown by the indirect antibody labeling and the receptor-blocking experiments, and the combination of the PDA with Tf could be used to actively target the B16F10 mouse melanoma cells in vitro, because of the upregulated Tf receptors on the surface of cancer cells. Through LIT analyses, PDA/Tf NPs were shown to convert the near-UV-light into heat, more efficiently, than the PDA/HSA. Both NPs were taken up by the J774A.1 mouse macrophages and the B16F10 cells. However, in the latter cell type, more PDA/Tf hybrid NPs were observed, intracellularly, than the PDA/HSA particles. This could be attributed to the fact that B16F10 cells are cancer cells. After exposure of the NPs to the B16F10 and J77A4.1 cells, we were able to induce apoptosis by irradiation of a defined region, with UV-light. The effect was more pronounced when irradiating with a UV-light source than when using a 633 nm laser, which was in line with the absorption spectra of the NPs. Additionally, the PDA hybrid, core-shell NPs were tested in an organotypic melanoma model, i.e., a 3D-printed spheroid, showing, again, a more pronounced induction of apoptosis, in the presence of the PDA/Tf NPs, when irradiated with 405 nm. Evidence suggests that LMP is the prevalent mechanism by which the PDA NPs can elicit an apoptosis response. We, thus, provide a new, highly adaptable system to target cancer cells and to induce apoptosis, via LMP, which is also capable of being effective in densely-packed cellular structures, like spheroids. By the combination of PDA with proteins, which are both inherently biocompatible, the system is unlikely to cause problems in organisms but this still needs in vivo experimental validation. If the results follow the expectations, the system has the potential to be used as a new cancer treatment. The inexpensive and straight-forward combination of the PDA with protein-based-targeting functionality, provides a suitable system for the further exploration of phototherapy, as the NPs can easily be modified and tailored to a specific task.

By targeting a basic cellular mechanism, namely the lysosomal degradation pathway, the NPs presented in this study, potentially, circumvent the development of resistances. However, a number of challenges need to be addressed to effect successful translation. For instance, the required UV stimulus has a very limited penetration depth, although the experiments with the spheroids showed a pronounced induction of apoptosis, also in the central layers. Systems that are capable of being stimulated by a longer wavelength radiation, might become accessible with a further investigation into how deep-cell-killing can be induced in the various progression states of the melanoma tumor tissues, as similar NPs have already been shown to undergo a stimulation with infrared light [27]. In conclusion, we have provided a promising and adaptable approach to combining the functionality of a protein or peptide with UV-light-induced heat generation, thereby, developing a potent tool to further explore the phototherapeutic applications of carbon-based functional materials.

## Figures and Tables

**Table 1:** Size and ζ-potential of the (PDA)/(has), PDA/Tf, and PDA/Tf-A488.

NP type	DLS in H_2_O (nm)	ζ-Potential in H_2_O (mV)	ζ-Potential in RPMI (mV)	ζ-Potential in DMEM (mV)
PDA/HSA	73.3 ± 0.5	−22.5 ± 1.5	−6.6 ± 1.5	−10.1 ± 3.0
PDA/Tf	30.0 ± 14.0	−28.6 ± 1.4	−12.5 ± 1.3	−5.9 ± 1.3
PDA/Tf-A488	244.6 ± 25.1	−25.1 ± 1.5	−5.1 ± 1.2	−7.8 ± 2.4

## Supplementary Materials

The following are available online at http://www.mdpi.com/2079-4991/8/12/1065/s1: Figure S1: Linear regressions for the absorbance, at 280 nm, of the different concentrations of Tf and HSA. Figure S2: Time-resolved DLS measurements of the PDA/HSA NPs and the PDA/Tf NPs, in DMEM, as well as the PDA/HSA NPs and the PDA/Tf NPs, in RPMI. Figure S3: Visualization and semi-quantification of the TfR in the J774A.1 mouse macrophages, B16F10 mouse melanoma cells, and monocyte-derived macrophages (MDMs), via Western blot. Figure S4: Immunofluorescent staining of the TfR in J774A.1 mouse macrophages and B16F10 mouse melanoma cells. Figure S5: Immunofluorescence staining of the PDA/HSA and the PDA/Tf NPs in the B16F10 mouse melanomas and the J774A.1 mouse macrophages. Figure S6: Uptake of the PDA/Tf-A488 NPs by the J774A.1 mouse macrophages, visualized by live cell imaging, via LSM, at different time points. Figure S7: Cytotoxicity assays of the PDA/Tf NPs on the B16F10 and the J774A.1. Figure S8: Cytotoxicity assays of the PDA/HSA NPs on the B16F10 and the J774A.1. Figure S9: Lysotracker^®^ colocalization with anti-Tf antibody. Figure S10: Analysis of the cell-death rate of spheroids subjected to either 405 nm laser light or 633 nm laser light, without the presence of NPs. Video S1: Uptake of the PDA/Tf-A488 NPs by the J774A.1 mouse macrophages. Video S2: J774A.1 mouse macrophages exposed to the PDA/HSA NPs. Light-induced cell-killing experiment. Video S3: Light-induced cell-killing experiment with the J774A.1 mouse macrophages in the absence of NPs. Video S4: B16F10 mouse melanoma cells exposed to the PDA/Tf NPs. Light-induced cell-killing experiment. Video S5: B16F10 mouse melanoma cells exposed to the PDA/HSA NPs. Light-induced cell-killing experiment. Video S6: Light-induced cell-killing experiment with the B16F10 mouse melanoma cells, in the absence of NPs. Video S7: Light-induced cell-killing experiment with the B16F10 mouse melanoma cells exposed to the PDA/Tf NPs, in the presence of Lysotracker^®^ lysosomal staining. Video S8: Light-induced cell-killing experiment with the B16F10 mouse melanoma cells, in the absence of NPs, in the presence of Lysotracker^®^ lysosomal staining.
