# Supplementary material for: Polydopamine/Transferrin Hybrid Nanoparticles for Targeted Cell-Killing
Source: Nanomaterials (Basel). 2018 Dec 17;8(12):1065. doi: 10.3390/nano8121065 (PMC6315732; doi:10.3390/nano8121065)
Supplement: Supplementary file 1 [file nanomaterials-08-01065-s001.zip › Supplementary_Hauser.pdf]

nanomaterials

MDPI

Supporting Information

Polydopamine / Transferrin Hybrid Nanoparticles for Targeted Cell Killing

Daniel Hauser[1], Manuela Estermann[1], Ana Milosevic[1], Lukas Steinmetz[1], Dimitri Vanhecke[1], Dedy Septiadi[1], Barbara Drasler[1], Alke Petri-Fink[1], Vincent Ball[2,3], Barbara Rothen-Rutishauser[1,*]

[1] Adolphe Merkle Institute, University of Fribourg, Chemin des Verdiers 4, 1700, Fribourg, Switzerland;
[2] Université de Strasbourg, Faculté de Chirurgie Dentaire, 8 rue Sainte Elisabeth, 67000, Strasbourg, France
[3] Institut National de la Santé et de la Recherche Médicale, Unité Mixte de Recherche 1121, 11 Rue Humann, 67085, Strasbourg Cedex, France

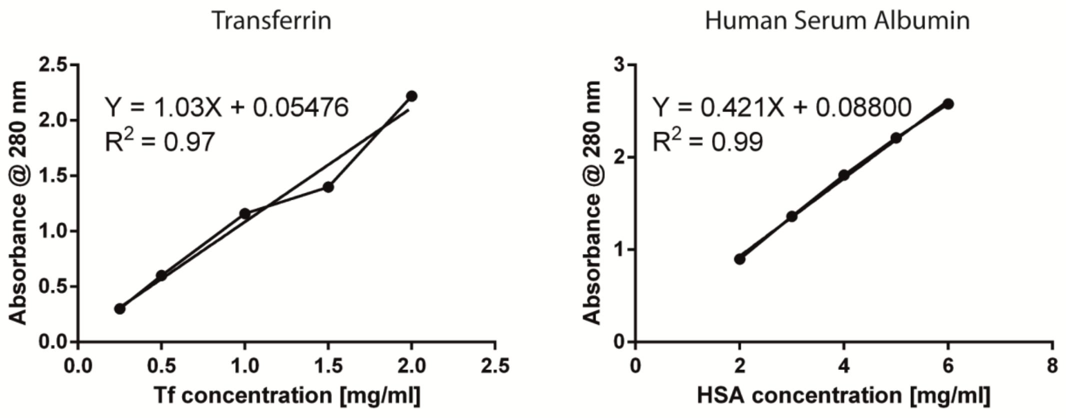

Supplementary Figure 1. Linear regressions for the absorbance at 280 nm of different concentrations of Tf (left) and HSA (right). The protein and PDA content of the NPs shown in Figure 1 were estimated using these graphs.

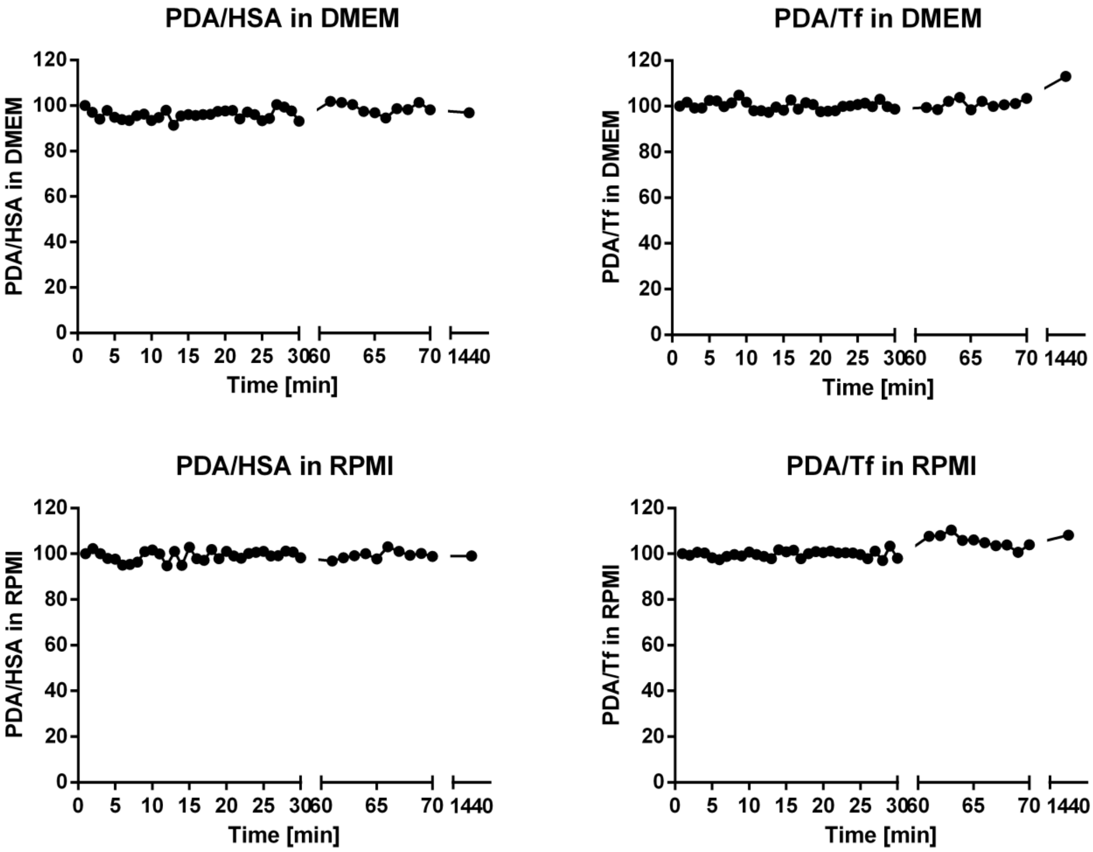

Supplementary Figure 2: Time-resolved DLS measurements of PDA/HSA NPs (A) and PDA/Tf NPs (B) in DMEM as well as PDA/HSA NPs (C) and PDA/Tf NPs in RPMI. The measured sizes were normalized whereby the first measured size is defined as 100%.

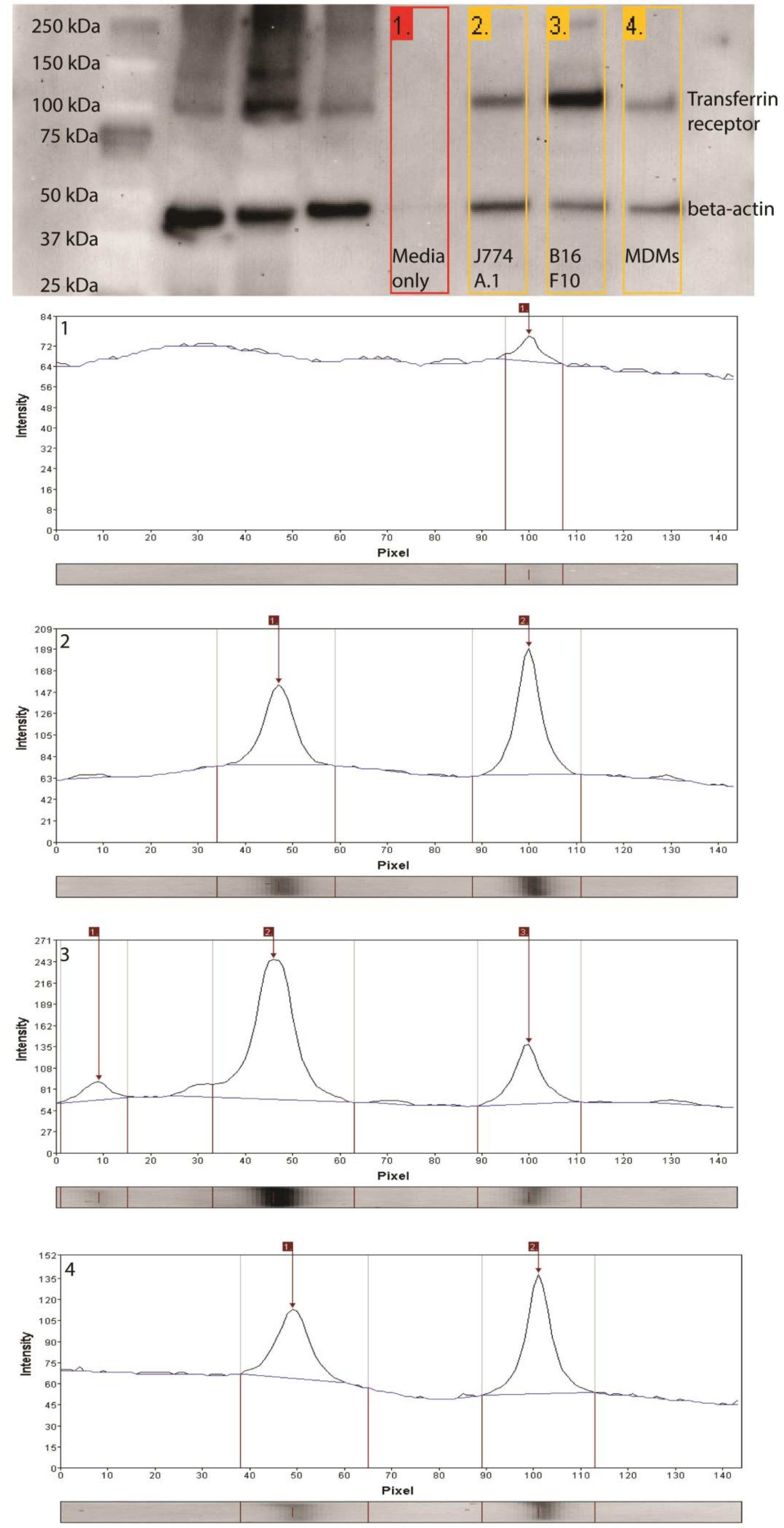
250 kDa
150 kDa
100 kDa
75 kDa
50 kDa
37 kDa
25 kDa
1.
2.
3.
4.
Transferrin receptor
beta-actin
Media only
J774 A.1
B16 F10
MDMs
Intensity
Pixel

Supplementary Figure 3. Visualization and semi-quantification of TfR in J774A.1 mouse macrophages, B16F10 mouse melanoma cells and monocyte-derived macrophages (MDMs) via Western blot. The WB shows protein lanes before (left side of the membrane) and after (right side of the membrane) concentration adjustment (50 μg). In the media control (graph 1) only beta actin is detected, while for J774A.1 mouse macrophages (graph 2), B16F10 mouse melanoma cells (graph 3) and primary macrophages derived from human buffy-coat-isolated monocytes (graph 4), TfR is also present. The TfR signal intensity (98 kDa) was further normalized by intensity of the beta-actin band for each cell type. The highest signal intensity/amount of TfR was detected in B16F10 mouse melanoma cells. More TfR was found in J774A.1 mouse macrophages than MDMs, but overall significantly less than in B16F10 mouse melanoma cells. The graphs' peaks appear in order as they appear on the WB membrane (from left to right corresponds to top to bottom).

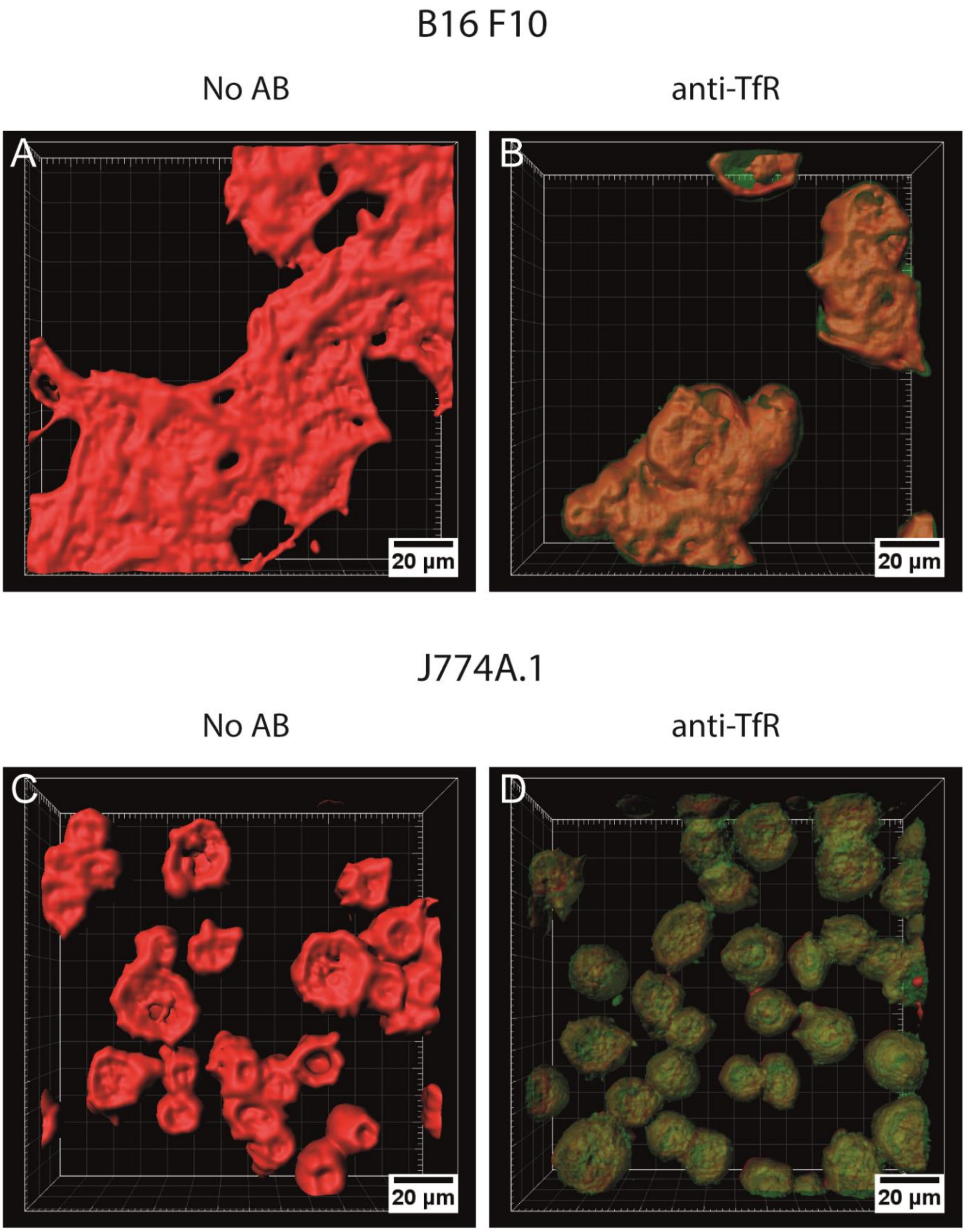

Supplementary Figure 4. Immunofluorescent staining of TfR in J774A.1 mouse macrophages and B16F10 mouse melanoma cells. Cells were stained for DNA (blue), F-actin (red) and TfR (green) and the images were rendered using the Imaris software. The TfR signal was found in and around the cells (B, D) when compared to the negative control (A, C), indicating its presence in the used cell types. The data was surface-rendered in imaris.

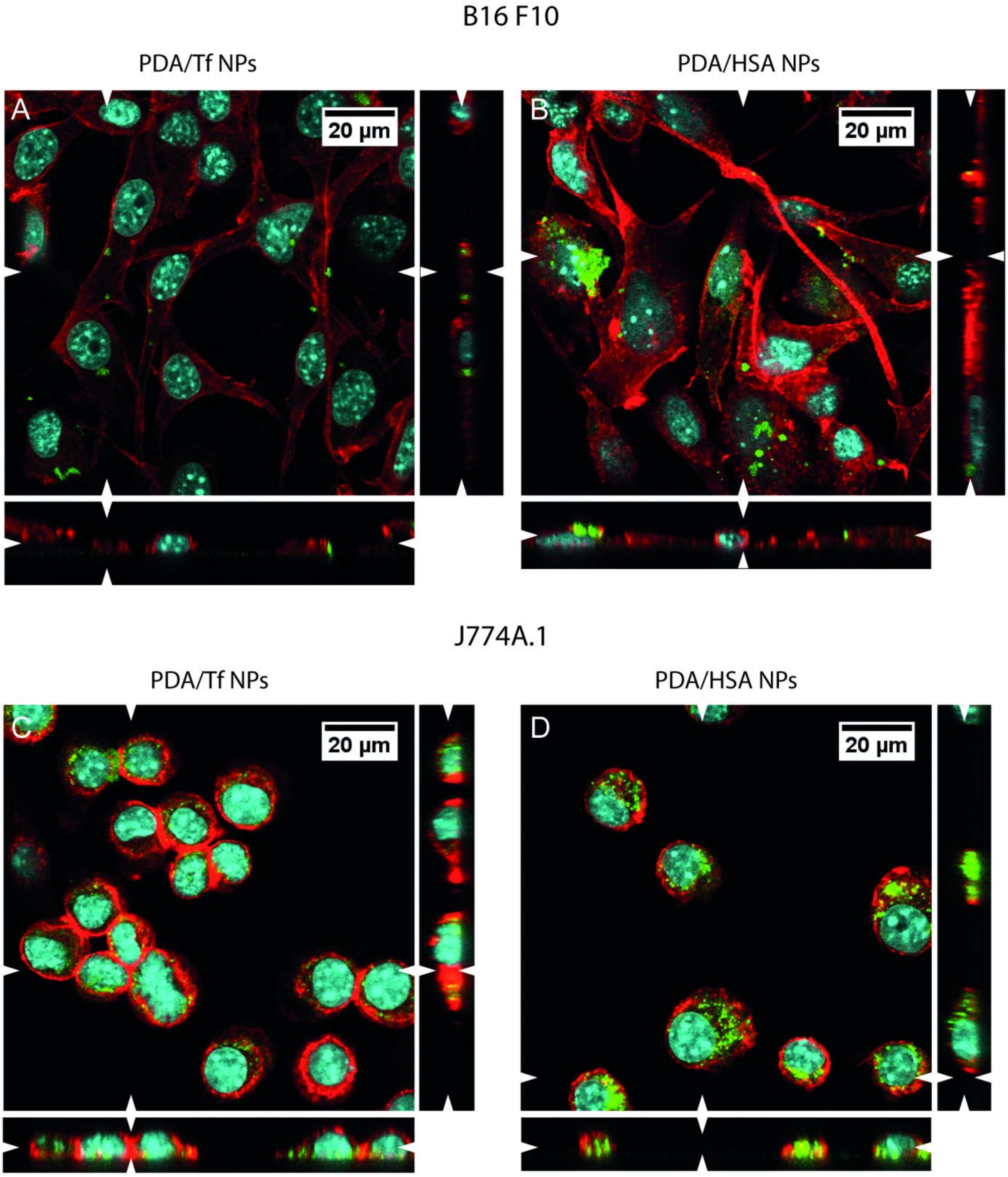

Supplementary Figure 5. Immunofluorescence staining of B16F10 mouse melanomas and J774A.1 mouse macrophages. B16F10 mouse melanoma cells and J774A.1 mouse macrophages were exposed to 20 µg/mL PDA/HSA or PDA/Tf for 24 h. Subsequently, the cells were fixed and the NPs were made visible via the addition of antibodies against either HSA or Tf (green). The F-actin (red) and nuclei (blue) were also stained. B16F10 exposed to PDA/Tf and anti-Tf antibodies, as well as that exposed to PDA/HSA NPs and anti-HSA antibodies, show signals inside the cell (A, B). Macrophages also showed intracellular signals under the same conditions (C, D). For better visualization, YZ and XY projections are depicted for each condition. Arrows indicate the cutting plane for projections.

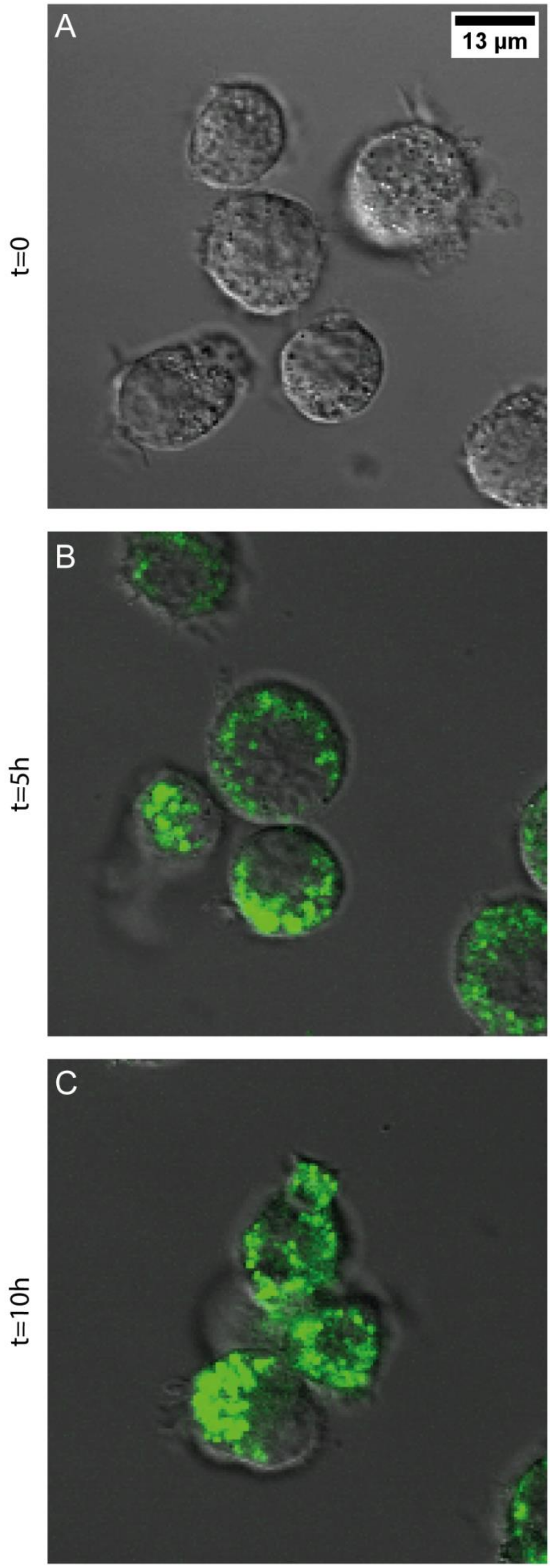
A
13 µm
B
C
t=0
t=5h
t=10h

Supplementary Figure 6. Uptake of PDA/Tf-A488 NPs (green) by J774A.1 mouse macrophages visualized by live cell imaging via LSM at different time points, i.e. 0 h, 5 h and 10 h. Within several hours a strong fluorescence signal could be detected within the cells. See also Supplementary Movie 1.

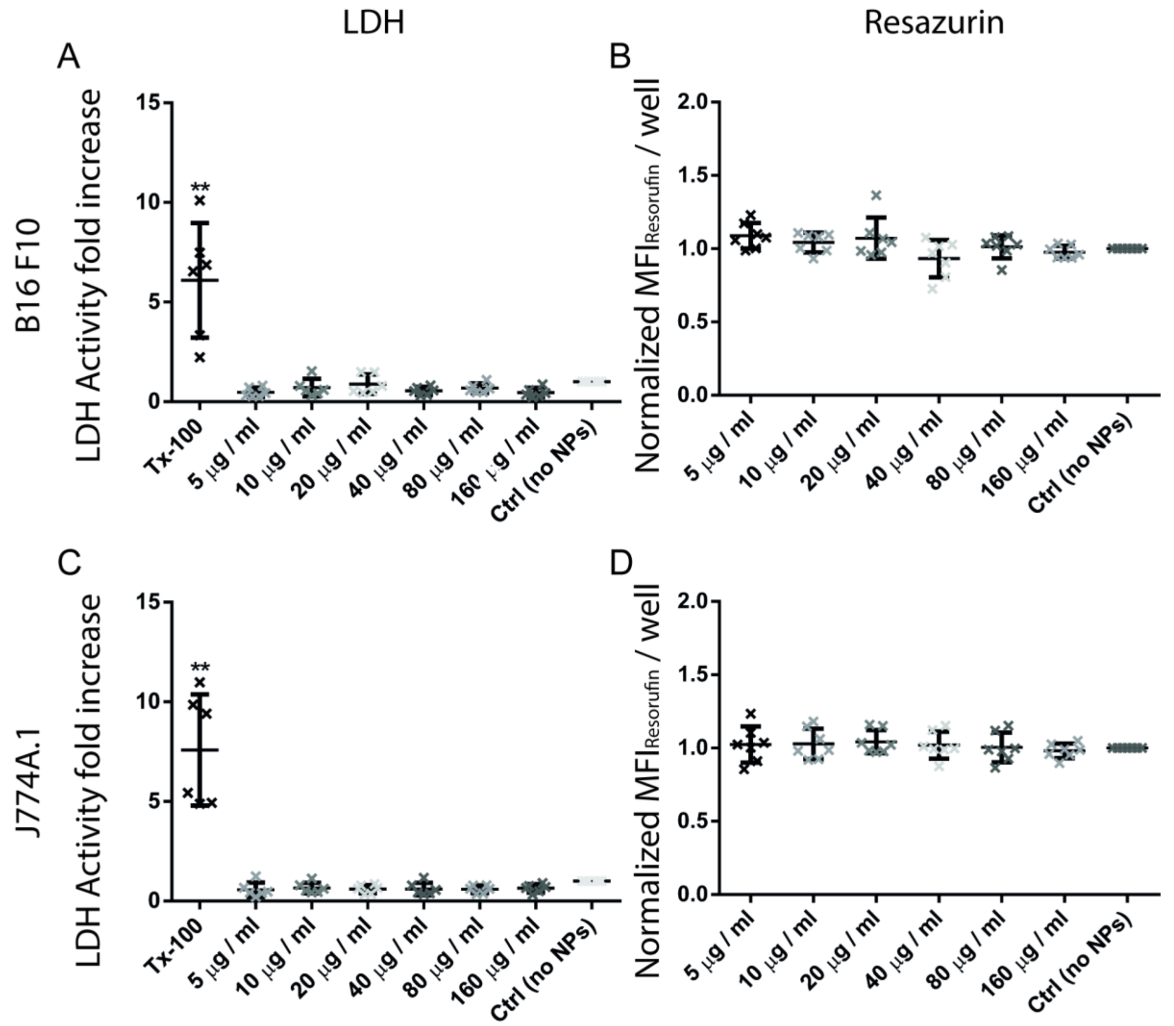

Supplementary Figure 7. Cytotoxicity assays of PDA/Tf NPs on B16F10 (A and B) and J774A.1 (C and D). Both LDH (A and C) and resazurin (B and D) approaches were conducted in order to obtain complimentary data. No cytotoxicity was found in either assay, while the positive control was significantly different from the exposed wells. "**": $p<0.01$, $n=6$.

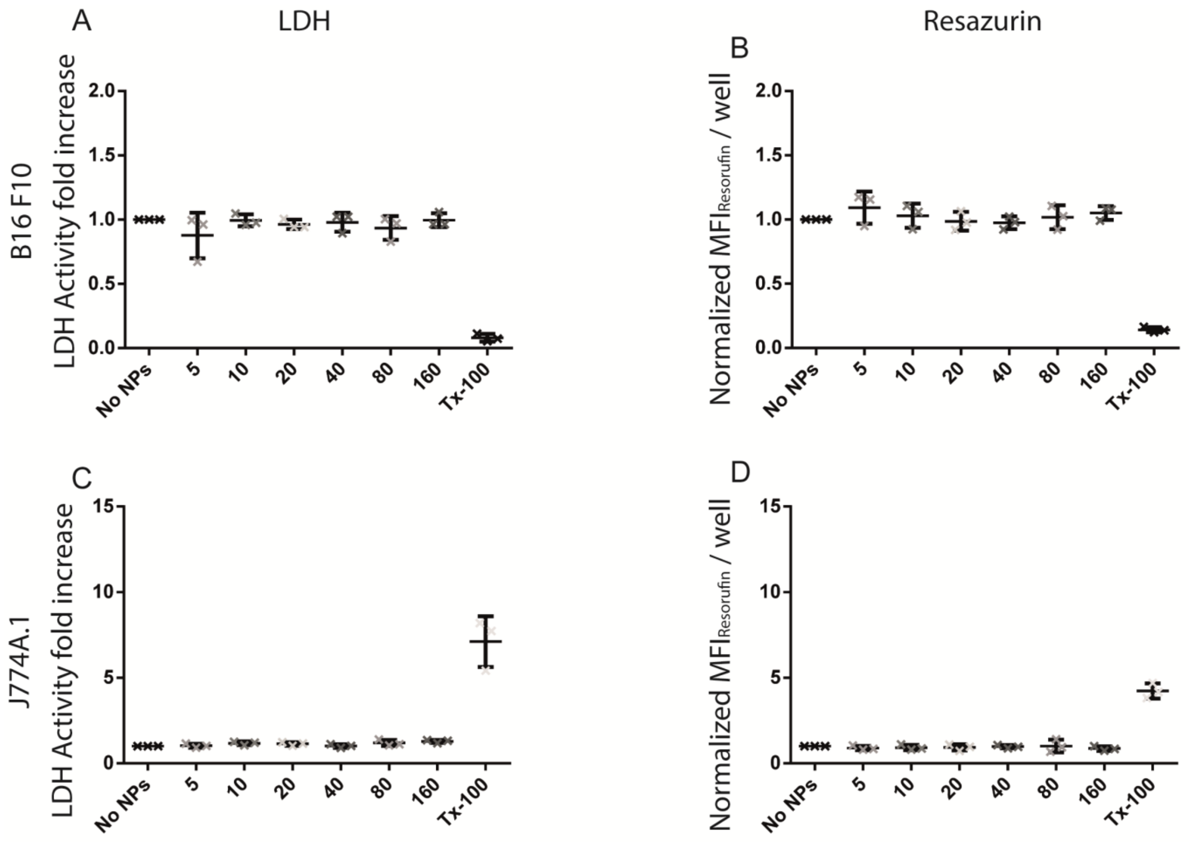

Supplementary Figure 8. Cytotoxicity assays of PDA/HSA NPs on B16F10 (A and B) and J774A.1 (C and D). Resazurin data for J774A.1 cells (A) and B16F10 cells (B) as well as LDH data for J774A.1 (C) and B16F10 (D). No cytotoxicity was observed for any tested concentration.

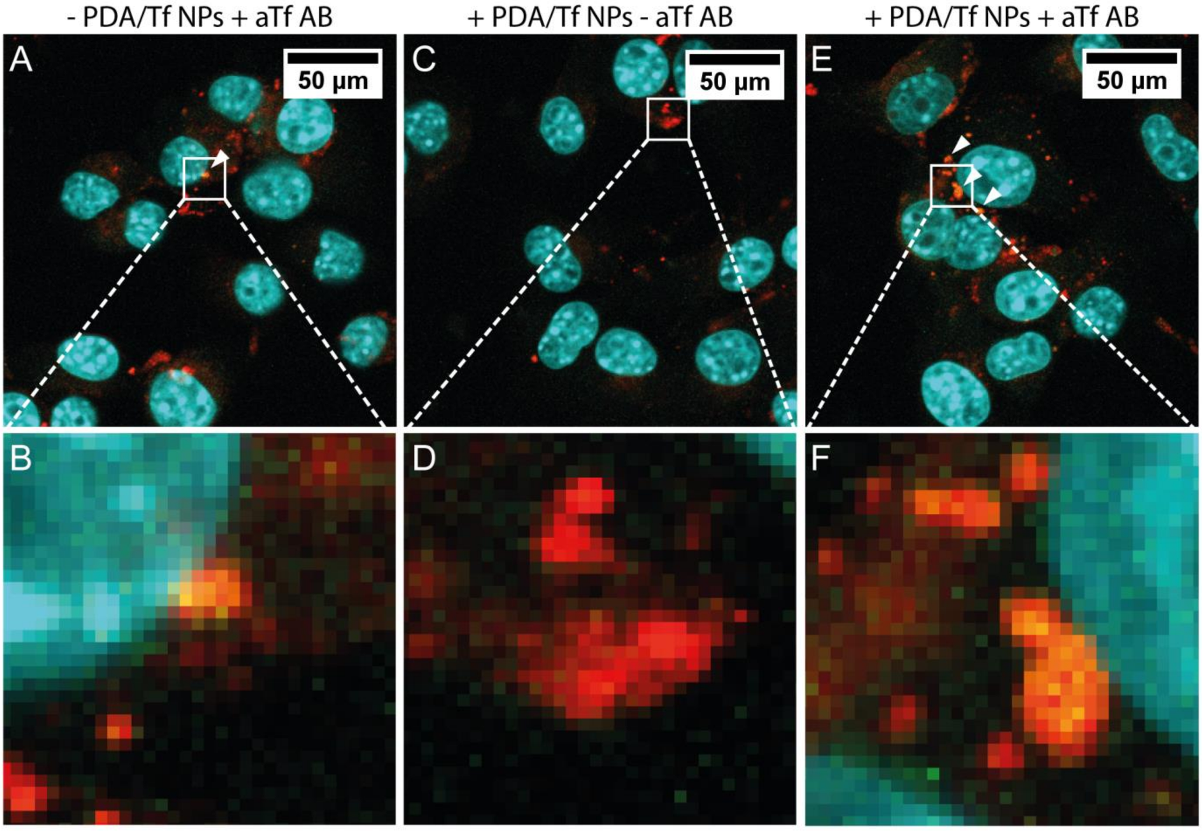

Supplementary Figure 9. cLSM image of Lysotracker® (red) in B16F10 mouse melanoma cells, anti Tf antibody (green) and Dapi (cyan). Panel A shows that there is also Tf present in the media, as some co-localization can be observed without any NPs present. Panel C does not show any co-localization. Panel E shows a higher percentage of co-localization between Lysotracker® and anti-Tf antibody. Panels B, D and F are magnifications of selected areas to visualize the colocalization (yellow). Pearson's R was calculated to be 0.24 for panel A, 0.05 for panel B and 0.38 for panel C, indicating a higher correlation when the PDA/Tf NPs are present.

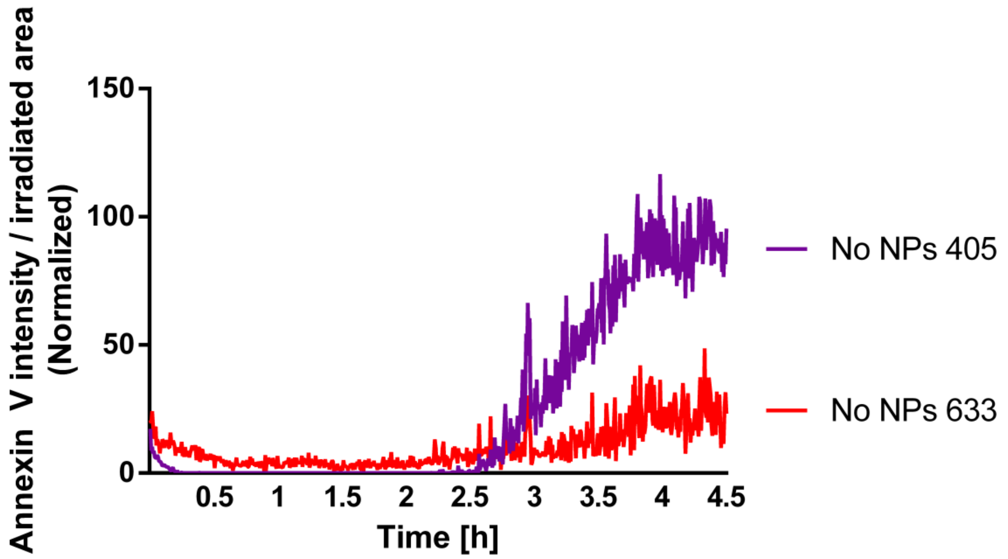

Supplementary Figure 10. Analysis of the cell death rate of spheroids subjected to either 405 nm laser light (violet) or 633 nm laser light (red) without the presence of NPs. N = 1.

Supplementary movie descriptions

- **Movie 1.** Uptake of PDA/Tf-A488 NPs by J774A.1 mouse macrophages.
- **Movie 2.** J774A.1 mouse macrophages exposed to PDA/HSA NPs. Light-induced cell killing experiment.
- **Movie 3.** Light-induced cell killing experiment with J774A.1 mouse macrophages in the absence of NPs.
- **Movie 4.** B16F10 mouse melanoma cells exposed to PDA/Tf NPs. Light-induced cell killing experiment.
- **Movie 5.** B16F10 mouse melanoma cells exposed to PDA/HSA NPs. Light-induced cell killing experiment.
- **Movie 6.** Light-induced cell killing experiment with B16F10 mouse melanoma cells in the absence of NPs.
- **Movie 7.** Light-induced cell killing experiment with B16F10 mouse melanoma cells exposed to PDA/Tf NPs in the presence of Lysotracker® lysosomal staining.
- **Movie 8.** Light-induced cell killing experiment with B16F10 mouse melanoma cells in the absence of NPs in the presence of Lysotracker® lysosomal staining.
